# Neonatal exposures to sevoflurane in rhesus monkeys alter synaptic ultrastructure in later life

**DOI:** 10.1016/j.isci.2022.105685

**Published:** 2022-11-30

**Authors:** Tristan Fehr, William G.M. Janssen, Janis Park, Mark G. Baxter

**Affiliations:** 1 Nash Family Department of Neuroscience and Friedman Brain Institute, Icahn School of Medicine at Mount Sinai, New York, NY 10029, USA; 2 Section on Comparative Medicine, Department of Pathology, Wake Forest University School of Medicine, Winston-Salem, NC 27157, USA

**Keywords:** Developmental neuroscience, Cellular neuroscience

## Abstract

Repeated or prolonged early life exposure to anesthesia is neurotoxic in animals and associated with neurocognitive impairment in later life in humans. We used electron microscopy with unbiased stereological sampling to assess synaptic ultrastructure in dorsolateral prefrontal cortex (dlPFC) and hippocampal CA1 of female and male rhesus monkeys, four years after three 4-h exposures to sevoflurane during the first five postnatal weeks. This allowed us to ascertain long-term consequences of anesthesia exposure without confounding effects of surgery or illness. Synapse areas were reduced in the largest synapses in CA1 and dlPFC, predominantly in perforated spinous synapses in CA1 and nonperforated spinous synapses in dlPFC. Mitochondrial morphology and localization changed subtly in both areas. Synapse areas in CA1 correlated with response to a mild social stressor. Thus, exposure to anesthesia in infancy can cause long-term ultrastructural changes in primates, which may be substrates for long-term alterations in synaptic transmission and behavioral deficits.

## Highlights

• Neonatal anesthesia alters hippocampus and prefrontal cortex synapses years later
• Anesthesia’s long-term impacts vary by synapse target, complexity, and region
• Mean synapse area in CA1 negatively correlates with altered socioemotional behavior
• In general, male and female monkeys were affected similarly by anesthesia

## Graphical abstract

**Figure:**
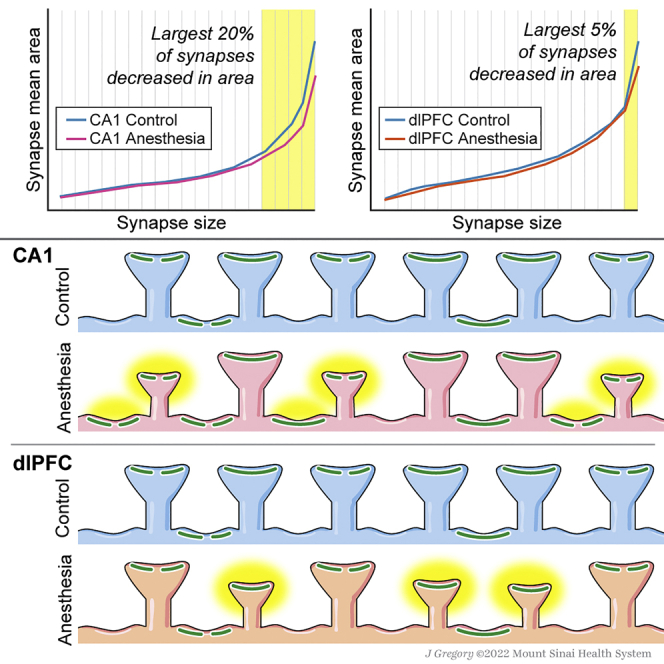

## Introduction

An estimated 500,000 to 1 million children under age 3 in the US are exposed to general anesthesia every year.^1^ Children exposed to anesthesia early in life are at greater risk for developing learning disabilities,^2^^,^^3^^,^^4^^,^^5^ socioemotional behavioral problems,^1^^,^^6^^,^^7^ and attention deficit-hyperactivity disorder.^8^^,^^9^^,^^10^^,^^11^ However, other studies have found little to no effect of early life anesthesia on different neurocognitive outcomes,^4^^,^^6^^,^^12^^,^^13^^,^^14^^,^^15^ supporting the hypothesis that anesthesia exposure early in life has domain-specific impacts.^16^

Nonhuman primates offer a number of advantages compared to rodents as a model for investigating how anesthesia exposures impact the developing brain and later life behaviors. Newborn rhesus macaques are at a stage of brain development similar to 6-month-old human infants,^17^ whereas rodent neurodevelopment in the first postnatal week is more comparable to human fetuses in the third trimester.^18^ Even though both species are susceptible to anesthetic neurotoxicity after birth,^19^^,^^20^ this reflects very different neurodevelopmental stages in both species. Rhesus macaques also display complex cognition and social behaviors that are ethologically relevant to human infants.^21^^,^^22^^,^^23^ Furthermore, anesthesia is safer to administer to nonhuman primates than to rodents because nonhuman primate physiology enables enhanced monitoring and support of physiological processes such as anesthetic and blood gas concentrations, pulse, blood pressure, and temperature.

Long-term behavioral outcomes of early life exposure to anesthesia have been recapitulated in rhesus monkeys. Rhesus monkeys exposed to anesthesia as infants show cognitive deficits^24^^,^^25^^,^^26^^,^^27^ and altered socioemotional behaviors^28^^,^^29^^,^^30^ in subsequent years. These observations suggest that repeated or prolonged exposure to general anesthesia early in life is sufficient to cause long term neurocognitive deficits.

The mechanism of such long-term neurocognitive deficits may relate to neuro- and gliotoxic effects of general anesthesia early in development.^19^^,^^31^^,^^32^^,^^33^ In general, neurotoxic effects of neonatal anesthesia in nonhuman primates have been shown on a short-term scale of hours to weeks following anesthesia exposure,^34^ though a recent study reported elevated GFAP expression and astrogliosis two years after exposure to isoflurane in infant rhesus monkeys.^35^ A pressing question remains regarding the neurobiological disturbances triggered by early life exposure to anesthesia that persist into the long-term. Such changes may be targets for strategies to remediate or improve neurocognitive function in cases where deficits are associated with general anesthesia early in life.

Exposure of rodents to anesthesia in infancy causes a deterioration of synaptic signaling, as evidenced by suppressed long-term potentiation (LTP),^32^^,^^36^^,^^37^^,^^38^^,^^39^ inhibited vesicular exocytosis,^40^ and altered expression of vesicular docking proteins.^41^^,^^42^ Neonatal anesthesia exposures in rodents alter the overall density of synapses in the hippocampus and subiculum,^43^^,^^44^^,^^45^ reduce large mushroom spines^38^ associated with perforated synapses and LTP,^46^ and drive proliferation of smaller immature spines and filopodia.^47^^,^^48^^,^^49^^,^^50^ These synaptic changes after anesthesia exposure in infancy are a potential mechanism for behavioral disturbances later in life.

Ongoing dysfunction of mitochondria is another possible mechanism linking early life anesthesia exposure and behavioral impairments. Early life anesthesia exposure in rodents reduces mitochondrial density and degrades mitochondrial ultrastructure in the hippocampus and subiculum.^45^^,^^51^^,^^52^ These changes are due in part to an elevation in reactive oxidative species (ROS), which disrupts the fission and fusion processes^53^ that recycle damaged mitochondria and compensate for their energetic dysfunction.^54^ The influx of ROS and the related depolarization of mitochondrial membranes by anesthesia^55^^,^^56^ can trigger mitochondria morphogenesis from tubular into abnormal vase and donut shapes.^57^^,^^58^ Donut and other degraded mitochondria contribute to the ongoing generation of ROS.^59^ Protecting mitochondria from ROS not only prevents anesthesia’s harmful effects on mitochondria density and ultrastructure, but also prevents cognitive impairments caused by anesthesia exposure.^52^^,^^60^ Abnormal mitochondrial count and shapes within presynaptic boutons could indicate chronic mitochondrial dysfunction related to deficits in synaptic transmission and behavior.

We investigated synapse and mitochondria structure using electron microscopy in postmortem brain tissue from monkeys that were tested longitudinally as part of our investigation of cognitive and socioemotional behavior after repeated postnatal sevoflurane exposure.^25^^,^^26^^,^^28^ These monkeys were exposed to sevoflurane three times for 4 h each, during the first, third, and fifth postnatal weeks, or underwent brief maternal separation on the same schedule. Monkeys were euthanized and tissue prepared for electron microscopy at the age of ∼4 years. We focused on hippocampal CA1 and layer III of the dorsolateral prefrontal cortex (dlPFC). We chose CA1 of the hippocampus because impairments in visual recognition and disrupted socioemotional behaviors follow neonatal hippocampal lesions,^61^^,^^62^ and layer III of the dlPFC because the development and function of dlPFC neurons depend on hippocampal integrity.^63^^,^^64^

## Results

### Quantification of synaptic ultrastructure and mitochondrial morphology

**Figure 1:**
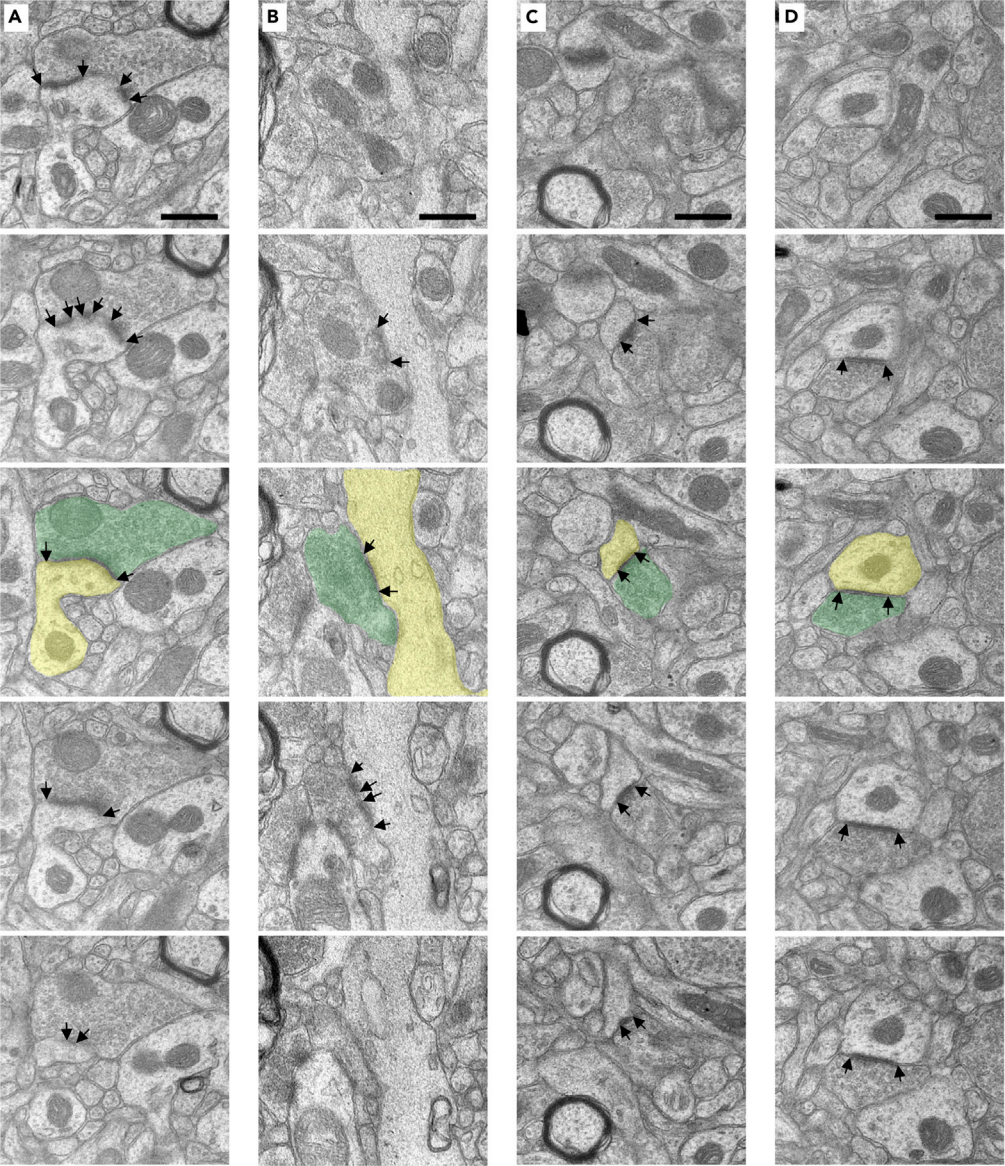
Examples of synapse ultrastructure by target and complexity (A–D) Left to right, serial sections of a perforated spinous (A), perforated dendritic (B), nonperforated spinous (C), and nonperforated dendritic synapse (D). Black arrows denote the boundaries of the synapse. Serial sections are 70 nm thick. Third section overlays indicate presynaptic (green) and postsynaptic (yellow) compartments. Scale bars are 500 nm.

**Figure 2:**
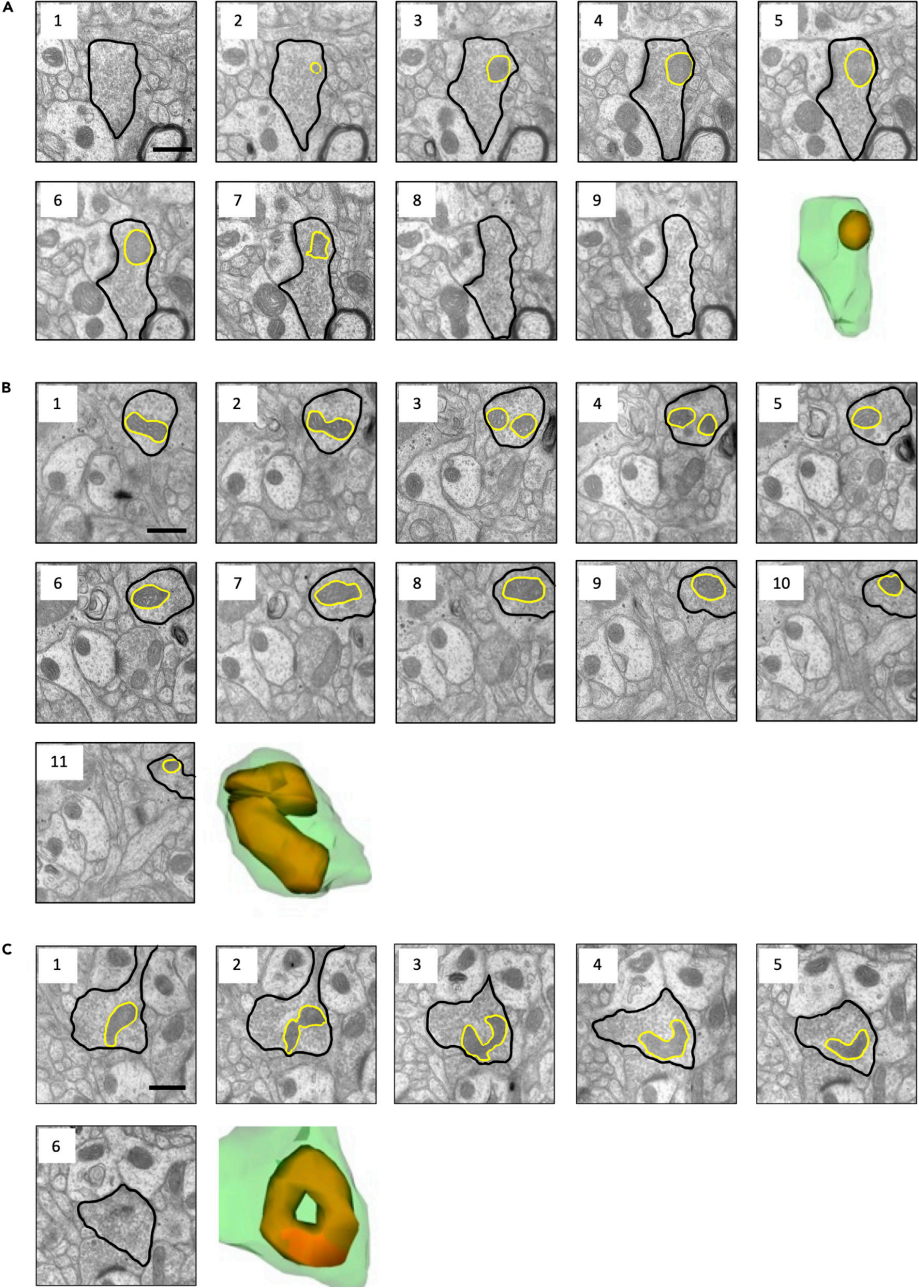
Examples of mitochondria ultrastructure (A–C) Serial electron microscope images and 3D reconstructions of a straight (A), curved (B), and donut (C) mitochondrion. Curved mitochondria had at least one ≤90° bend, donut mitochondria formed ring shapes, and all other mitochondria were classified as straight. In electron microscope images, presynaptic bouton is outlined in black and mitochondrion is outlined in yellow. In reconstructions, presynaptic bouton is green and mitochondrion is orange. Serial sections are 70 nm thick. Scale bars are 500 nm.

We used electron microscopy to identify synaptic contacts in ultrathin (70 nm) tissue sections from stratum radiatum of the CA1 field of the hippocampus and layer III of dlPFC. Sections were cut in serial ribbons of 15–20 sections to permit stereological quantification approaches. All electron microscopy imaging, image processing, and image analysis was conducted by an experimenter blind to experimental condition and sex of the monkeys. Synapses were identified by presynaptic vesicles and a synaptic cleft and classified by their postsynaptic target: Dendritic spines (spinous synapses) or dendritic shaft (dendritic synapses); and synapse complexity: perforated morphology or nonperforated (macular or continuous) morphology. Perforated synapses were identified by discontinuity in the synapse. Dendritic spines were identified by their ovoid shape, often with a neck and spine apparatus; dendritic shafts were identified by presence of microtubules, and often mitochondria. Examples of perforated spinous (Figure 1A), perforated dendritic (Figure 1B), nonperforated spinous (Figure 1C), and nonperforated dendritic (Figure 1D) synapses are shown. We assessed the morphology and number of mitochondria within presynaptic boutons across all 5 of the 15-section series per animal. Mitochondria within each presynaptic bouton were classified as straight, curved, or donut-shaped (toroidal) and counted (Figures 2A–2C). All analyses used individual animals (cases) as the basic statistical unit.

### Early life anesthesia exposures reduce areas of the largest CA1 and dlPFC synapses, but do not affect synapse density or vesicle docking

**Figure 3:**
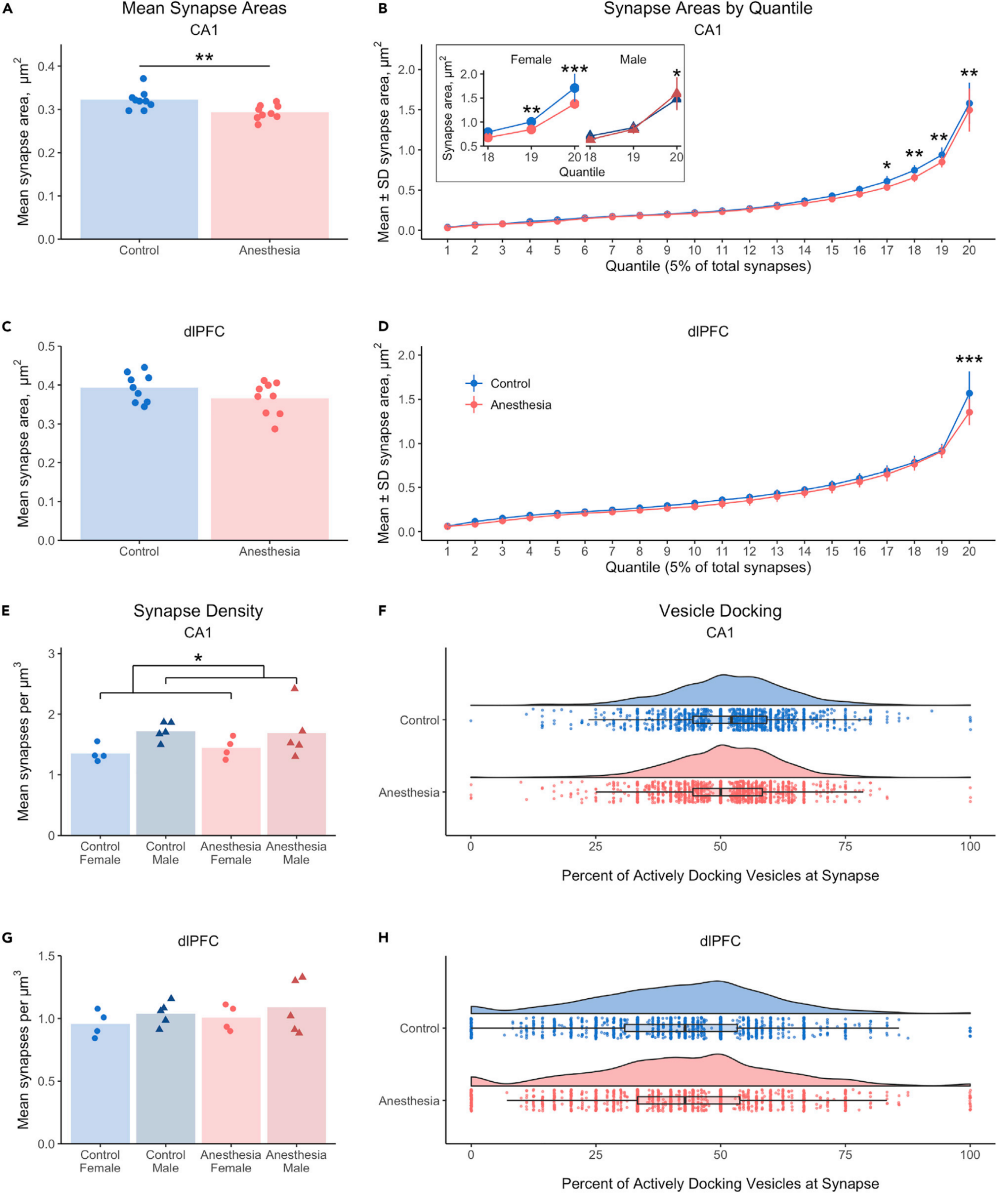
Early life anesthesia exposures reduce areas of the largest CA1 and dlPFC synapses, but do not affect synapse density or vesicle docking (A) Mean synapse areas were reduced 8.9% in CA1 of monkeys exposed to anesthesia as infants (p < 0.006). (B) In CA1, the largest 20% of synapse areas were smaller in monkeys exposed to anesthesia, shown in the 17^th^ (p = 0.02), 18^th^ (p < 0.004), 19^th^ (p = 0.003), and 20^th^ quantiles (p < 0.001). (B *inset*) Females had lower synapse areas associated with anesthesia exposure in the 19^th^ (p < 0.005) and 20^th^ quantiles (p < 0.0001), and males had higher synapse areas in the anesthesia group versus controls in the 20^th^ quantile (p = 0.036). (C) Mean synapse area was not affected by anesthesia or sex in dlPFC. (D) A treatment by quantile interaction in the dlPFC resulted from a reduction in area of the largest 5% of synapses (20^th^ quantile) in monkeys exposed to anesthesia as infants (p < 0.0001). (E) Synapse density in CA1 was 22% greater in males than females (p = 0.03), but was not affected by anesthesia. (F) The proportion of docking vesicles in CA1 synapses was not significantly impacted by early-life exposure to anesthesia. (G) Synapse density in dlPFC was not affected by anesthesia, sex, or their interaction). (H) The proportion of docking vesicles in dlPFC synapses was not affected by anesthesia. (A–H) Unexposed controls shown in blue, monkeys exposed to anesthesia shown in red. (A, C, E, and G) Individual points indicate individual monkey means. (B and D) Data points indicate pooled group means per quantile, ±SEM (B *inset*, E, and G) Females are lighter shades with circles, males are darker shades with triangles. (F and H) Kernel distribution graphs show the relative spread of the frequency of areas, box & whisker plots show the range and quartiles of areas, and individual points are individual synapses. ∗ = p < 0.05, ∗∗ = p < 0.01, ∗∗∗ = p < 0.001, LMM. Complete statistical results are presented in Table S1.

We hypothesized that repeated exposure of monkeys to the common pediatric anesthetic sevoflurane, in three 4-h sessions over the first five postnatal weeks, would result in altered synapses and mitochondria within CA1 of the hippocampus and the dlPFC four years after exposure. First, we evaluated the long-term effects of anesthesia on synapse size by measuring synapse areas. In the CA1, monkeys exposed to anesthesia as infants showed an overall 8.9% reduction in mean synapse area compared to controls (control 0.32 ± 0.02, anesthesia 0.29 ± 0.02 μm^2^, Figure 3A). To determine whether this effect varied per synapse area—e.g., if larger synapses were more vulnerable—we sorted synapse areas in ascending order and divided the resulting lineup for each animal into 20 bins (quantiles) that each comprised 5% of the total number of their synapse area measurements. Pairwise comparisons of control and anesthesia groups showed synapses of monkeys in the anesthesia group were significantly reduced in the 17^th^–20^th^ quantiles Figure 3B). This effect was moderated by sex in the 19^th^ and 20^th^ quantiles (Figure 3B, inset), where anesthesia-exposed males but not females showed relative sparing of synapse area relative to their respective controls.

Atypically large nonperforated synapses, defined as synapses with areas greater than two standard deviations above the mean, exhibit unique excitatory receptor expression profiles compared to typical nonperforated synapses in the CA1.^65^ To determine the long-term effects of early life anesthesia on atypically large nonperforated synapses, we measured the numbers and areas of atypically large nonperforated synapses in monkeys exposed to anesthesia and controls. Monkeys exposed to anesthesia had significantly fewer atypically large nonperforated spinous synapses in CA1 (p = 0.03), but these did not differ in their areas compared to control monkeys, p = 0.72. No differences in atypically large nonperforated synapse number or area were found between anesthesia and control monkeys for nonperforated dendritic synapses in CA1 (there were only 16 total in the entire sample, 8 in the anesthesia group and 8 in the controls).

In the dlPFC, we found no significant overall effects of treatment or sex on mean synapse area (Figure 3C). However, when synapse areas were sorted into 20 quantiles, a significant treatment by quantile interaction showed a reduced synapse area in the largest 5% of synapses, with synapse areas 13.6% smaller in monkeys exposed to anesthesia as infants compared to controls (1.57 ± 0.25 control, 1.35 ± 0.15 μm^2^ anesthesia, Figure 3D). No differences in synapse number or area were found for atypically large nonperforated spinous or dendritic synapses in the dlPFC (p > 0.05). Therefore, only the largest synapses in the dlPFC showed long-term impacts of anesthesia exposure in infancy.

We did not find significant effects of anesthesia treatment or treatment by sex interactions on CA1 synapse density, but males showed a 22% greater synaptic density compared to females (Figure 3E). Synapse density in dlPFC did not show any effects of treatment or sex (Figure 3G). We also measured the proportion of docking vesicles in anesthesia and control monkeys as a proxy for baseline synaptic activity. The proportions of docking vesicles were not significantly affected by anesthesia treatment, sex, or their interaction in CA1 or dlPFC (Figures 3F and 3H). Thus differences in synapse area in CA1 and dlPFC related to early anesthesia exposure are not accompanied by changes in overall synapse density or the availability of vesicles proximate to the synapse.

### Early life anesthesia differentially affects synapses classified by target and complexity

We next asked whether these changes were specific to synapse type based on the predominance of differences in synapse area in the largest fractions of synapses in both regions. Axodendritic synapses tend to be larger than axospinous synapses in the CA1 of humans^66^ and perforated synapses with complex shapes are larger on average than nonperforated synapses with simple disc shapes,^67^ so we classified synapses based on target (“spinous” and “dendritic”) and complexity (perforated versus nonperforated).

**Figure 4:**
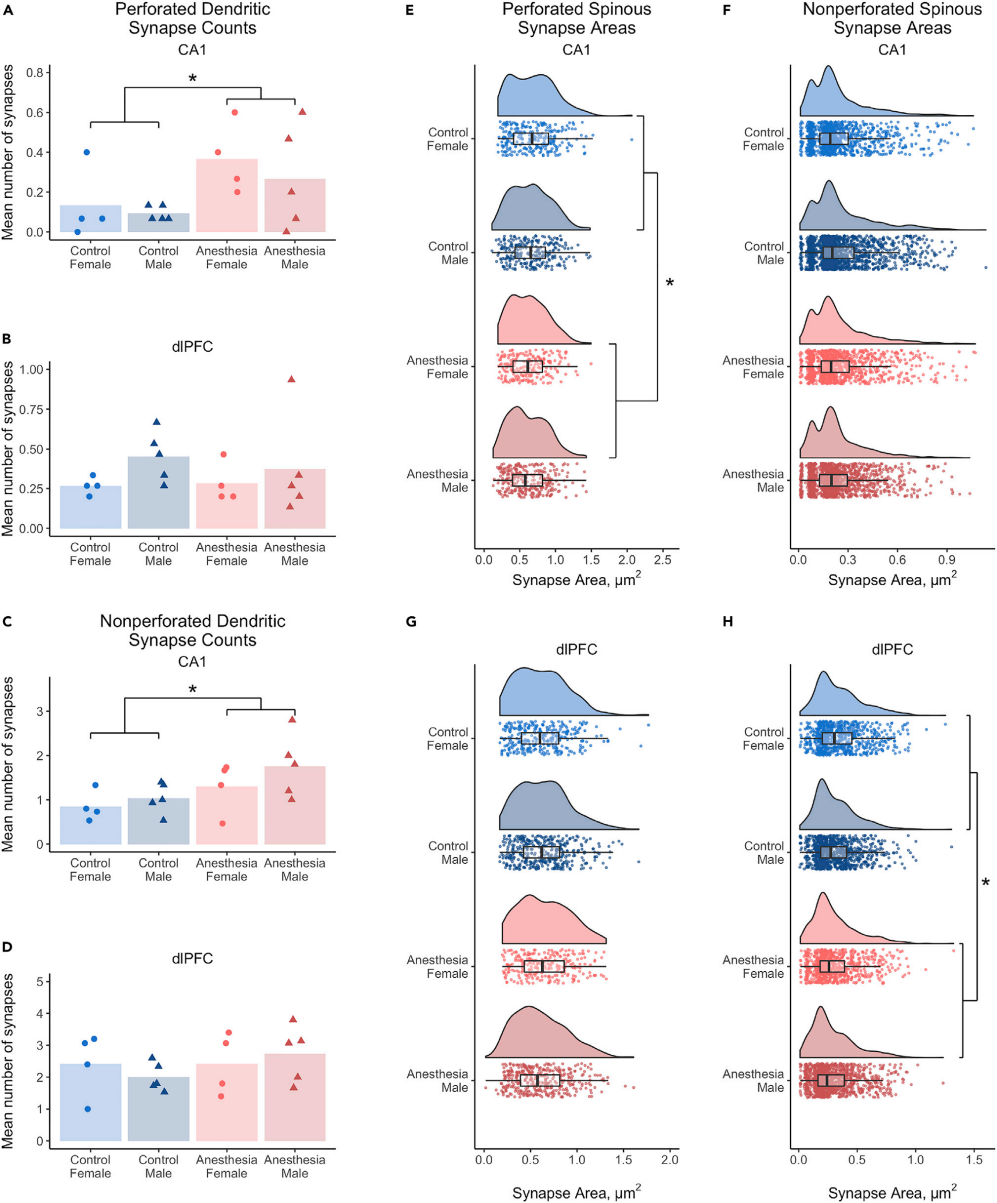
Early-life anesthesia differentially affects synapses classified by target and complexity (A) Monkeys exposed to anesthesia as infants had 180% more perforated dendritic synapses in CA1 than unexposed controls (p = 0.013). (B) Numbers of perforated dendritic synapses in dlPFC were not affected by anesthesia. (C) Monkeys exposed to anesthesia as infants had 63% more nonperforated dendritic synapses in CA1 than unexposed controls (p = 0.014). (D) Numbers of nonperforated dendritic synapses in dlPFC were not affected by anesthesia. (E) Perforated spinous synapse areas in CA1 were 7.5% smaller in monkeys exposed to anesthesia in infancy compared to unexposed controls (p = 0.042). (F) Areas of nonperforated spinous synapses in CA1 were not affected by anesthesia. (G) Perforated spinous synapse areas in dlPFC were not affected by anesthesia. (H) Areas of nonperforated spinous synapses in dlPFC were 10.4% smaller in monkeys exposed to anesthesia in infancy compared to controls (p = 0.021). (A–D) Individual points indicate individual monkey means. (E–H) Kernel distribution graphs show the relative spread of the frequency of areas, box & whisker plots show the range and quartiles of areas, and individual points are individual synapses. Colors and symbols are as in Figure 3. ∗ = p < 0.05, χ^2^ (A–D), LMM (E–H). Complete statistical results are presented in Table S1.

Early life exposures to anesthesia caused a long-term, selective increase in numbers of dendritic synapses in CA1 but not dlPFC. Monkeys exposed to anesthesia as infants had more perforated and nonperforated dendritic synapses in CA1 compared to controls (Figures 4A and 4C), whereas synapse numbers by subclass did not differ in dlPFC (Figures 4B and 4D). Spinous synapses were smaller in both regions, an effect limited to perforated spinous synapses in CA1 and nonperforated spinous synapses in dlPFC (Figures 4E–4H).

### Long-term changes in mitochondrial morphology and localization were subtle

**Figure 5:**
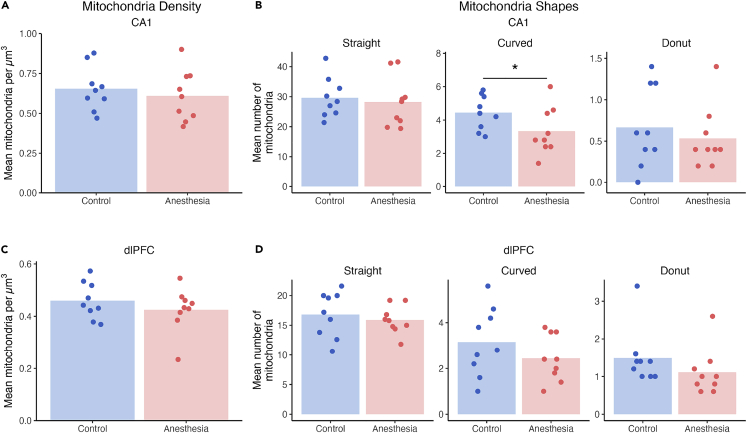
Early life exposure to anesthesia selectively affected mitochondria shapes but not overall density mitochondria (A) In CA1, the overall density of mitochondria in presynaptic boutons was not affected by exposure to early life anesthesia, sex, or their interaction. (B) Monkeys exposed to anesthesia in infancy showed a 25% reduction in curved mitochondria in CA1 compared to unexposed controls (p = 0.042), and no differences in straight or donut mitochondria. (C) In dlPFC, the density of mitochondria in presynaptic boutons was not affected by early life anesthesia, sex, or their interaction. (D) Exposure to anesthesia in early life did not alter numbers of straight, curved, or donut mitochondria. Unexposed controls shown in blue, monkeys exposed to anesthesia shown in red. Individual points indicate individual monkey means. ∗ = p < 0.05, χ^2^. Complete statistical results are presented in Table S2.

The numbers of mitochondria in presynaptic boutons per cubic micron of tissue were not affected by treatment, sex, or their interaction (Figures 5A and 5C). Mitochondrial health and maintenance, as indicated by their straight, curved, or donut-shaped morphology, was different only in CA1, where monkeys that were exposed to anesthesia as infants had 25% fewer curved mitochondria in presynaptic boutons compared to controls (Figure 5B). In the dlPFC, we found no effect of treatment, sex, or their interaction on mitochondria morphology (Figure 5D). In summary, exposures to anesthesia in infancy imparted a region-specific, long-term reduction in curved mitochondria in CA1 presynaptic boutons.

**Figure 6:**
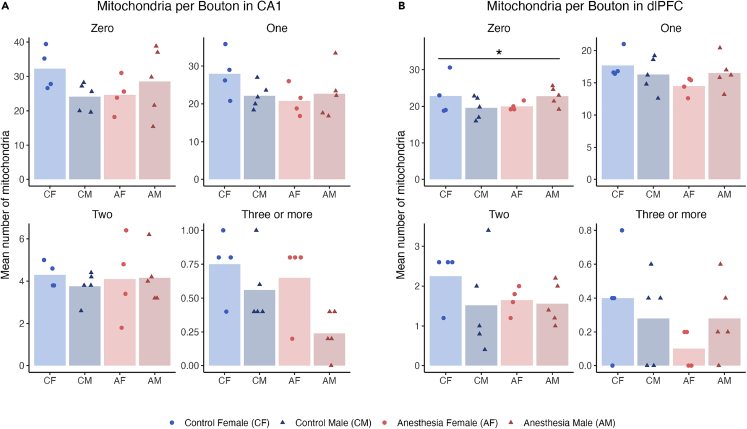
Early life anesthesia exposure only significantly altered the numbers of mitochondria per presynaptic bouton in the dlPFC (A) The numbers of mitochondria per presynaptic bouton in CA1 showed a trend toward a significant interaction of early life anesthesia exposure with sex (p = 0.09), and trends toward the interaction of anesthesia and sex for boutons with 0 mitochondria (p = 0.07), and sex for boutons with 3+ mitochondria (p = 0.05). (B) The numbers of mitochondria per presynaptic bouton in dlPFC showed a significant interaction of early life anesthesia exposure with sex (p = 0.013), with an interaction of early life anesthesia with sex for boutons with 0 mitochondria (p = 0.042), and a trend toward an interaction effect of anesthesia and sex for boutons with 1 mitochondrion (p = 0.088). Unexposed controls shown in blue, monkeys exposed to anesthesia shown in red. Females are lighter shades with circles, males are darker shades with triangles. Individual points indicate individual monkey means. ∗ = p < 0.05, χ^2^, for individual categories. Complete statistical results are presented in Table S2.

We also assessed how early life anesthesia affected the relative abundance of mitochondria within presynaptic boutons by measuring the numbers of presynaptic boutons containing 0, 1, 2, and 3 or more mitochondria. There were no statistically significant differences related to anesthesia treatment on these measures in CA1 (Figure 6A). In the dlPFC, the numbers of mitochondria per presynaptic bouton showed a treatment by sex interaction across categories, driven by a significant treatment by sex interaction in boutons with 0 mitochondria (Figure 6B). Boutons with 0 mitochondria were decreased by anesthesia relative to controls in females, and increased in males.

### Synapse area in CA1 negatively correlates with socioemotional behaviors

The monkeys in our cohort that were exposed to anesthesia in infancy exhibited increased emotional reactivity to a social stressor, as was shown in the human intruder test throughout their infancy and adolescence.^25^^,^^26^ To assess whether the behavioral changes shown by monkeys exposed to anesthesia as infants are associated with changes in synaptic ultrastructure, we assessed the correlation between each monkey’s mean synapse area and an aggregate *Z* score of behaviors in the human intruder test. We found a moderate negative correlation between mean synapse area in the CA1 and human intruder test z-scores (r(16) = −0.54, p = 0.021). We found no significant correlations of human intruder z-scores with mean synapse area in the dlPFC, in quantiles 19 or 20 for CA1 or dlPFC, or with perforated spinous area in CA1 or nonperforated spinous area in dlPFC (p > 0.05). These results demonstrate that a reduction in mean CA1 synapse area is associated with an increase in social stress-induced behaviors.

## Discussion

Monkeys exposed to anesthesia multiple times as infants showed region-specific and sometimes sex-specific alterations in their synaptic ultrastructure 4 years later compared to unexposed monkeys. These findings support the presence of long-term changes in synaptic ultrastructure after exposure to general anesthesia in infancy in primates, lasting at least until adolescence. The negative correlation of mean synapse area in the CA1 with socioemotional behaviors in the human intruder test suggests that the enduring synaptic deficits instated by anesthesia could represent a substrate for the changes in memory and socioemotional behavior we observed in these monkeys—functions that depend on the integrity of the hippocampus and connected structures. These data support exploring approaches to remediate synaptic health after early anesthesia exposure to treat neurobehavioral deficits.

### Long-term effects of anesthesia exposure on synapses

The loss of spinous synapse area in monkeys exposed to sevoflurane in infancy is consistent with effects of early life anesthesia on spines in rodents. Spinous synapse lengths were reduced in CA1, 3–5 months after multiple neonatal sevoflurane exposures in male rats,^43^ aligning with our observations of CA1 vulnerability to changes in synapse area by anesthesia exposure. By contrast, synapse lengths were not affected in rat subiculum 2 weeks after a single anesthetic exposure (Lunardi et al. 2010), suggesting potential regional effects of anesthesia exposures as well as the importance of elapsed time since exposure for the manifestation of synaptic changes. Spine head volumes correlate highly with synapse area,^68^ and decrease in average size in rodent hippocampus and cortex after early life anesthesia.^47^^,^^48^^,^^49^ This decrease is characterized in mice by both an outgrowth of smaller, labile spines and filopodia^50^ and the loss of large, stable mushroom spines.^38^^,^^69^^,^^70^ The reduction in synapse areas that we found in the largest synapses in CA1 and dlPFC in absence of an overall change to synapse density suggests that a similar shift in spine morphology could persist years after anesthesia in nonhuman primates.

Likewise, the reduction in spinous synapse areas in CA1 and dlPFC and reduction in the number of atypically large nonperforated spinous synapses in the CA1 of monkeys exposed to neonatal anesthesia imply an enduring loss of excitatory synapse coverage. Almost all (91-99%) spinous synapses in the CA1 and cortex of rodents and primates are excitatory.^66^^,^^71^^,^^72^^,^^73^^,^^74^^,^^75^^,^^76^ In adult rat CA1, 100% of perforated and 64% of nonperforated synapses contain AMPA receptors, and 100% of both subtypes contain NMDA receptors.^67^^,^^77^ Similarly, atypically large nonperforated synapses in adult rat CA1 contain more AMPA and NMDA receptors than typical nonperforated spinous synapses (Nicholson & Geinisman 2009). These expression patterns indicate that the reduction in synapse areas and number of atypically large nonperforated spinous synapses that we found in monkeys could translate to AMPA and NMDA receptor loss, a plausible substrate for reduced excitatory postsynaptic potentials^44^^,^^78^ and suppressed LTP after neonatal anesthesia exposure in rodents.^32^^,^^36^^,^^37^^,^^38^^,^^39^

The increase in perforated and nonperforated synapses targeting CA1 dendrites that we found in monkeys has more ambiguous implications. In rats, global reductions in synapse density have been reported shortly after anesthesia exposure in rodents, with reduced axospinous synapse density found 3–5 months after single and multiple exposures starting on P7^43^, and decreases in glutamatergic synapse density reported two months after a single exposure on P7.^79^ However, these studies did not distinguish the effects of anesthesia on dendritic synapses alone. One study in rats that did differentiate anesthesia’s effects on dendritic synapses contrasted with our finding, showing that a neonatal exposure to an anesthetic cocktail caused no difference in the ratio of mean spinous to dendritic synapse numbers in the subiculum two weeks later.^45^ However, subtle changes to dendritic synapses could be harder to detect in rats because synapses targeting dendrites comprise only 5% of the total synapses in rat CA1 ^72^ compared to 21% in primate (human) CA1.^67^

### Long-term effects of anesthesia exposure on mitochondria

The lack of long-term effects of anesthesia on overall mitochondrial density in CA1 and dlPFC presynaptic boutons in monkeys contrasts with the acute and long-term modulation of mitochondria density by neonatal anesthesia in the subicular cytoplasm^51^^,^^53^ and CA1 presynaptic terminals of rats.^43^ The distinction between these findings could be because of methodological differences, including and not limited to interspecies differences in neurodevelopmental timelines, dose and type of anesthetics used, sex, and regions analyzed. Regardless, the lack of effect of anesthesia exposure on the overall mitochondria density in monkey CA1 and dlPFC suggests that mitochondrial density is not grossly and enduringly impaired in these regions.

By comparison, the reduction in numbers of curved mitochondria by anesthesia exposure in the monkey CA1 may point to a disturbance in energy management processes managed by mitochondrial fusion and fission. Curved mitochondria can form through the fusion of healthy mitochondria with damaged mitochondria to compensate for dysfunctional components.^54^ This process unfolds over the course of postnatal development in rats.^80^ Consistent with the acute disruption of the fusion-fission axis by anesthesia in rats,^53^^,^^81^ the reduction in curved mitochondria we observed in monkeys could be a function of decreased fusion, excessive fission, or both.

The modulation of numbers of boutons with 0 mitochondria in dlPFC by early life anesthesia hints at additional long-term changes in neuronal responsivity to energy needs. Healthy neurons at rest use 4.7 billion ATP per second,^82^ and meet additional energy expenditure demands by recruiting mitochondria along filamentous actin.^83^ Presynaptic boutons without mitochondria are reliant on ATP diffusion and show less vesicle docking in response to theta-burst stimulation that produces LTP.^80^ This suggests that the fluctuation in the number of presynaptic boutons lacking mitochondria in the dlPFC in monkeys exposed to anesthesia as infants could translate to variance in the synaptic efficiency of dlPFC neurons, although the apparent modulation of this effect by sex requires further investigation.

### Regional dissociation of ultrastructural changes by anesthesia

The dissociation of early life anesthesia’s impacts on the CA1 and the dlPFC could be because of distinct windows of vulnerability to anesthetic insults. The regional nature of the ultrastructural changes we observed is unlikely to be because of gross differences in anesthetic neurotoxicity: Apoptosis of neurons and glia is widespread in nonhuman primates following early life exposure to anesthesia.^17^^,^^31^^,^^84^ However, synaptogenesis in nonhuman primates differs by region. Hippocampal structures such as the dentate gyrus undergo rapid synaptogenesis in the latter half of gestation, achieve adult levels of synaptic density by birth, and peak in density 4–5 months postnatally.^85^ By comparison, the rapid period of synaptogenesis in prefrontal cortex occurs from two months before to two months after birth.^86^ These distinct timelines for synaptogenesis in the nonhuman primate brain suggest offset windows of vulnerability for anesthesia’s effects on synapse development. Indeed, the interplay of region and age of exposure to neonatal anesthesia has been shown to differentially impact synapse number and maturity in rodents.^47^^,^^48^^,^^49^^,^^50^^,^^70^ Likewise, given that dlPFC neurons depend on hippocampal integrity for their development and function,^63^^,^^64^ disruption of the regions’ interaction by anesthesia could be another factor contributing to regional differences in synaptic ultrastructure later in life. Research that takes advantage of the variations in synaptic development timelines between brain regions could be a useful tool in elucidating both anesthesia’s actions and potential therapeutic targets.

### Connecting structural changes to behavioral deficits

We found a significant correlation between reduced mean synapse areas in the CA1 and altered socioemotional behaviors. These data suggest that the structural alterations caused by early life anesthesia are associated with changes in synaptic plasticity and efficiency that contribute to deficits in behavioral performance. In several mouse models of neurodevelopmental disorders, behavioral deficits have been linked to impaired LTP related to synaptic restructuring.^87^^,^^88^^,^^89^ Similarly, in mice exposed to ketamine as neonates, stressor-evoked anxiety-like behaviors in adulthood were linked to a deficit in LTP mediated by impaired AMPA receptor recruitment to synapses.^90^ Mitochondrial disturbances are related to impaired working memory in monkeys and recognition memory in rats,^91^^,^^92^ and to anxiety-like behaviors in a chronic social defeat paradigm in mice.^93^ Our study supports the role of synapse remodeling by anesthesia in generating behavioral impairments throughout later life in nonhuman primates, as well.

### Clinical relevance

Our results indicate that synaptic remodeling set into motion by early life anesthesia could be one basis for clinically significant behaviors in later life. Although the changes to synapse area that we report here might be viewed as modest, it is unclear that a near-10% reduction in synapse area in the hippocampus is dispensable or that most parents would view this as an acceptable potential side effect of surgery on their child. However, anesthesia in early life will remain a necessity for safe surgeries. A deeper understanding of the long-term neurobiological and behavioral risks associated with anesthesia can therefore allow for more informed clinical decision-making. Similarly, insights into the mechanisms behind the ultrastructural and behavioral changes that result from early life exposure to anesthesia can better direct the development of therapeutic interventions that can remediate those impacts, both at the time of exposure and into later life. Ultimately, uniting mechanistic knowledge with clinical solutions promises to help those who undergo anesthesia in early life experience full lives that are unfettered by anesthesia’s consequences.

### Methodological considerations and conclusions

Our anesthesia paradigm of three instances of 4 h of sevoflurane may pertain best to children at the high end of anesthesia exposure. Approximately 26% of the 500,000 to 1 million infants and children exposed to anesthesia every year have a single exposure greater than 3 h, or multiple exposures before age 3.^1^^,^^94^ This means that the results we report here may be most relevant to those tens of thousands of infants annually with the greatest cumulative anesthetic history. Yet, because single exposures to anesthesia have also been found to confer an increased risk for behavioral problems in children,^1^ the structural findings we report here may also lend themselves to the long-term outcomes of shorter exposures to anesthesia.

In addition, our experimental approach only addresses the effects of anesthesia exposure in early childhood, rather than in adolescence or adulthood. Clinically, cognitive and behavioral deficits have not been seen in children who were exposed to anesthesia after age 5.^95^^,^^96^ In line with human data, rats show a window of neurotoxic vulnerability specific to early postnatal development.^20^ Moreover, a single long anesthesia exposure does not incur cell death or disrupt neurogenesis in adult rats.^97^ The end of the window of neurotoxic vulnerability has not been observed in nonhuman primates, with infant macaques vulnerable to neurotoxicity until at least postnatal day 40.^19^ However, studies in adults and geriatric populations have focused in on inflammation rather than anesthesia as a mechanism for cognitive effects, given that no association has been found between time to cognitive recovery after anesthesia and age in healthy adults 40–80 years old.^98^ To date, there is little indication that anesthesia exposure, per se, constitutes a problem of similar gravity in adults.

To our knowledge, this is the first study in nonhuman primates to show that exposure to general anesthesia in infancy leaves a synaptic signature that endures for years. Our findings extend prior research on the effects of early life anesthesia on neuronal ultrastructure in rodents and nonhuman primates and demonstrate that effects of anesthesia on synaptic ultrastructure persisted into adolescence in monkeys. We propose that the ultrastructural changes that we found in monkeys with neonatal anesthesia exposure may be related to their impairments in visual recognition memory and heightened emotional reactivity to social stressors and may represent potential targets for therapeutic approaches.

### Limitations of the study

Although our sample size was large for ultrastructural research in monkeys, we were underpowered for evaluating sex-based effects, especially for modest effect sizes. We may have failed to detect more subtle treatment-by-sex effects, as suggested by trends toward significance in the numbers of dlPFC synapses by type and numbers of mitochondria in presynaptic boutons. To avoid confounding effects of subsequent anesthesia exposures, we avoided any experimental measures between anesthesia exposures and brain collection that would have required additional anesthesia, such as neuroimaging, electrophysiology, or additional tissue collection, limiting functional corroboration of our postmortem ultrastructural measures. Nonetheless, the ultrastructural changes we found four years after neonatal exposures to anesthesia provide unique insights into the long-lasting impacts of neonatal anesthesia on primate neurobiology. Further experimental validation will be needed to test whether synaptic changes have direct clinical significance, perhaps by determining biomarkers that can bridge the translational chasm between detailed ultrastructural analyses in animal models and less invasive approaches that can be applied in humans.

## STAR★Methods

### Key resources table

**Table:** 

REAGENT or RESOURCE	SOURCE	IDENTIFIER
**Biological samples**		
Rhesus macaque (*Macaca mulatta*)	Yerkes National Primate Research Center	N/A

**Chemicals, peptides, and recombinant proteins**		

Sevoflurane	Millipore Sigma	Cat#1612540
Ketamine hydrochloride	Millipore Sigma	Cat#1356009
Pentobarbital	Millipore Sigma	Cat#1507002
Paraformaldehyde	Electron Microscopy Sciences	Cat#19208
Glutaraldehyde	Electron Microscopy Sciences	Cat#16314
Sodium cacodylate	Electron Microscopy Sciences	Cat#11654
Osmium tetroxide	Electron Microscopy Sciences	Cat#19152
Uranyl acetate	Ted Pella, Inc.	Cat#19481
Lead citrate	Electron Microscopy Sciences	Cat#22410
Propylene oxide	Electron Microscopy Sciences	Cat#20412
EPON resin, EMbed-812 Kit	Electron Microscopy Sciences	Cat#14120

**Deposited data**		

Dataset and analysis code	This paper	https://doi.org/10.5281/zenodo.7272343

**Software and algorithms**		

Reconstruct	SynapseWeb	https://synapseweb.clm.utexas.edu/software-0; RRID:SCR_002716
SynBin	Adams et al. 2001	N/A
R	R Core Team, 2020	http://www.r-project.org/; RRID:SCR_001905
Adobe Photoshop	Adobe	Creative Suite 5; RRID:SCR_014199
DHARMa package for R	Hartig, 2022	https://cran.r-project.org/web/packages/DHARMa/; RRID:SCR_022136

**Other**		

Leica Vibratome VT1000S	Leica	RRID:SCR_016495
Diamond knife	Diatome	Ultra
Formvar-coated slot grids	Electron Microscopy Sciences	FCF2010-Ni
Hitachi H-7000 TEM microscope	Hitachi High Technologies America, Inc.	N/A
AMT Advantage 10 CCD camera	Advanced Microscopy Techniques	https://amtimaging.com

### Resource availability

#### Lead contact

Further information and requests for resources should be directed to and will be fulfilled by the lead contact, Mark Baxter (mgbaxter@wakehealth.edu).

#### Materials availability

This study did not generate new unique reagents.

### Experimental model and subject details

#### Rhesus macaques (*Macaca mulatta*)

All animal procedures were approved by the Yerkes National Primate Research Center and the Emory University Institutional Animal Care and Use Committee, and were conducted in full compliance with United States Public Health Service Policy on Humane Care and Use of Laboratory Animals. Rhesus monkeys were raised at Yerkes National Primate Research Center. All data was acquired from female and male monkeys at ∼48 months of age.

### Method details

#### Monkey anesthesia and behavior

Infant rhesus macaques in the anesthesia group (n = 5 female, n = 5 male) received a 4-h exposure to ∼2.5% sevoflurane on ∼ P7 (range P6-10), two weeks later at ∼ P21, and two weeks later again at ∼ P35 for a total of 3 anesthetic exposures between ∼ P7-35.^25^ Monkeys in the control group (n = 5 female, n = 5 male) received a brief maternal separation for ∼30 min following the same time schedule as the anesthesia group. Sample sizes were determined based on statistical power to detect behavioral effects of anesthesia exposure in a between-groups comparison.^25^ We did not specifically power the study for sex differences but chose to include equal numbers of males and females in each experimental group.

After removal from their dam, all infants received a brief neurological exam. At this point, anesthesia group monkeys were mask-induced with sevoflurane (from 2 vol % to effect, maximum 8 vol % in 100% O_2_), intubated, and catheterized for IV fluids. Sevoflurane was administered for 4 h (∼2.5% sevoflurane in oxygen balanced with medical air (30% O_2_), with monitoring of vital signs, depth of anesthesia and blood gases. Results of physiological monitoring during anesthesia were consistent with normal physiology, with no indications of hypoxemia, hypercapnia, or hypotension. Upon complete recovery usually within 20-30 min, the infant was returned to its dam. For control group subjects, the maternal separation procedure consisted of the neurological exam and a period of handling with a duration of separation that matched that of the period of conscious separation experienced by the experimental group. On average, control infants experienced 30-40 min of maternal separation, and were returned to their dam. Mother-infant interactions after these separations did not differ between groups, indicating that the separations involved in anesthesia exposure did not alter mother-infant bonding that might have impacted later cognitive or socioemotional behavior.^99^

At 6, 12, 24, and 48 months, monkeys were tested in a human intruder paradigm ^25^^,^^26^ and were also tested in a visual paired comparison test of recognition memory at 6, 12, and 24 months.^28^

#### Brain collection and preparation for electron microscopy

At ∼48 months of age (range 47.8–49.8 months, median age 49.5 months), monkeys were perfused. Monkeys were deeply anesthetized with ketamine hydrochloride (25 mg/kg) and pentobarbital (20–35 mg/kg, i.v.), intubated, and mechanically ventilated to prevent hypoxia and ischemia. Monkeys were transcardially perfused with cold 1% paraformaldehyde in 0.1 M phosphate buffer (PFA/PB; pH 7.2) for 2 min, followed by 4% (w/v) PFA and 0.125% glutaraldehyde/PB at 250 mL/min for 12 min.

After perfusion, tissue was postfixed for 6 h in 4% PFA and 0.125% glutaraldehyde/PB, washed in PB. Coronal blocks containing the dorsolateral prefrontal cortex or hippocampus were sectioned at 50- and 400-μm thicknesses on a vibratome (Leica) for light and electron microscopy, respectively. Specifically, 12 50-μm sections and one 400-μm section were taken in every mm of brain tissue, with a random placement of the first 400-μm section within the series of 50-μm sections to allow for stereologic sampling. From the 400-μm sections, we generated two sets of tissue blocks for EM analysis.

Tissue sections were blocked to identify hippocampal CA1 at a mid-anterior/posterior level from the body of the hippocampus posterior to the uncus, as well as the dlPFC spanning the principal sulcus (Brodmann’s area 46). Tissue was washed in sodium cacodylate buffer, placed in 1% osmium tetroxide/double distilled water (ddH2O) for 1 h, washed with ddH2O, and stained *en bloc* in cold 2% uranyl acetate/ddH2O for 1 h in the dark. After a ddH2O wash, tissue was dehydrated through an ascending ethanol/ddH2O series, washed 10 min in 1:1 ethanol:propylene oxide (PO), 10 min in PO, and infiltrated with EPON resin (Electron Microscopy Sciences, EMbed 812 Kit): ascending resin:propylene oxide with pure resin overnight and again the next morning. Blocks were embedded in BEEM capsules and placed in a vacuum for 72 h at 60°C.

In preparation for ultrastructural analysis, tissue from stratum radiatum of CA1 and layer III (250-350 μm from the layer I-II intersection) of dlFPC was semi-thin sectioned at 1 μm on a Leica UC-7 ultramicrotome, and stained with toluidine blue to further identify regions of interest for ultrathin (70 nm) serial sections. Ribbons of 15-20 ultrathin (70 nm) serial sections were cut using a Diatome diamond knife (Electron Microscopy Sciences), mounted on Formvar-coated slot grids (Electron Microscopy Sciences), and counter-stained with uranyl acetate and lead citrate.

#### Imaging on electron microscope

All electron microscopy imaging, image processing, and image analysis was conducted by an experimenter blind to experimental condition and sex of the monkeys. Monkeys were assigned a random alpha-numeric code for tracking purposes during imaging and image analysis that contained no information about their sex or anesthesia group. This code was broken only after all image analyses had been completed. One control female monkey and one anesthesia female monkey were excluded from further analysis due to an unresolvable possible confusion of their tissue blocks during initial processing. Thus, samples from 9 control monkeys (n = 5 male, n = 4 female) and 9 anesthesia monkeys (n = 5 male, n = 4 female) continued forward into further analysis.

Slot grids containing ribbons of at least 15 serial sections from the region of interest were imaged on a Hitachi H-7000 TEM microscope (Hitachi High Technologies America, Inc.) with an AMT Advantage CCD camera (Advanced Microscopy Techniques) at 12000x. Briefly, 5 non-overlapping image series of 15 sequential sections were captured per animal. Care was taken to avoid blood vessels, cell bodies, and post-staining artifacts in the image fields. Imaging was conducted in tandem with post-exposure image processing in Adobe Photoshop (CS5) to maximize field alignments, sharpness, and brightness between sections. Image field area was 83.09 μm^2^, and section thickness was 70 nm per section, for a section volume of 5.82 μm^3^. The total volume per 15-section series was 87.25 μm^3^.

### Quantification and statistical analysis

#### Image processing and quantitative analyses

Each image series of 15 sequential sections from the CA1 stratum radiatum or dlPFC layer III was imported into the open-source software Reconstruct (https://synapseweb.clm.utexas.edu/software-0) to create reconstructions of brain tissue for feature analysis in three dimensions. Section images were sequentially aligned to each other using the manual linear alignment tool beginning at the middle, or eighth, section and moving to either end of the 15-section series. CA1 and dlPFC measurements were tabulated separately.

#### Synapse areas

Synapses were identified by presence of presynaptic vesicles and a synaptic cleft. Each 15-section series was subdivided into 3 non-overlapping series of 5 sections. For each of these 5-section subseries, the lengths of synapses identified on the middle, or third, section were traced to completion across the sections using Reconstruct’s z-tracing tool. Because we had serial sections and could follow synapses across their entire extent, we used integrated area in our analyses rather than postsynaptic density length as a measure of synapse size. Synapse areas (μm^2^) were calculated by multiplying the sum of synapse lengths in μm by the total thickness of the sections they crossed (0.070 μm ∗ number of sections). Synapses that intersected the x- and y-limits of the sections were excluded from analysis. Areas of n = ∼250-330 synapses in dlPFC and n = ∼350-450 synapses in CA1 were calculated per animal.

#### Synapse area classifications by type

Synapses were also classified by their postsynaptic target: dendritic spines (spinous synapses) or dendritic shaft (dendritic synapses); and synapse complexity: perforated morphology or nonperforated (macular or continuous) morphology. Perforated synapses were identified by a discontinuity in the synapse. Dendritic spines were identified by their ovoid shape, often with a neck and spine apparatus; dendritic shafts were identified by presence of microtubules, and often, mitochondria. Once synapses were classified according to their targets and complexity, synapse areas and total synapse number in each category per 5-section series (29.08 μm^3^) were calculated. Examples of perforated spinous (Figure 1A), perforated dendritic (Figure 1B), nonperforated spinous (Figure 1C), and nonperforated dendritic (Figure 1D) synapses are shown.

#### Synapse density with optical disector

Synapse density was measured using an optical disector approach. 5 separate pairs of adjacent section images were selected from each of three 15-section series, for a total of 15 pairs per animal. For each pair of sections, only the synapses that were unique to each section and did not appear on the adjacent section were counted. The number of unique synapses at the leading edge in each section was divided by the section volume of 5.82 μm^3^ to calculate synapse density for n = 30 sections per animal.

#### Vesicle docking

We used measures of vesicle docking to estimate synaptic activity in our regions of interest. We used the program SynBin^100^ to define the boundaries of presynaptic boutons and the lengths of synapses, and to mark vesicles therein for quantification. Vesicles in axonal boutons are each ∼35 nm and docked vesicles are often smaller than docked vesicles.^68^ Therefore, we estimated that actively docking vesicles would appear within 30 nm of the presynaptic membrane, while vesicles that are 30-60 nm away from the membrane represent vesicles that are not actively docking. For each of 10 synapses per section, we counted the number of vesicles 0-30 nm and 30-60 nm from the inner edge of the presynaptic membrane, defined respectively as the docking and pre-docking zones. We calculated vesicle docking as the ratio of the number of docked vesicles to the sum of docked and pre-docked vesicles for each synapse. In each of the 5 series of 15 sections per animal, we performed analyses on 3 sections that were separated by at least 350 nm (5 sections) to avoid sampling the same synapses, for a total n = 150 synapses analyzed per animal per region and n = 2,700 total synapses analyzed per region.

#### Presynaptic mitochondria density, number per bouton, and morphology

We assessed the morphology and number of mitochondria within presynaptic boutons across all 5 of the 15-section series per animal. Presynaptic boutons were the focus of mitochondrial analyses at the synapse. To start, we identified presynaptic boutons transected by the middle, or eighth, section of each series by the presence of 3 or more synaptic vesicles within their enclosed membranes. We followed each bouton to completion across sections, up and down the z axis of the 15-section series. In order to not preferentially exclude large boutons, we included boutons that intersected the top (first) and bottom (15th) sections of the series in the analysis [large boutons comprise an estimated 6-14% of all boutons analyzed^101^]. Boutons that were intersected by the x and y axes were excluded from analyses. All measurements of mitochondria pertain only to those within the presynaptic boutons identified in this manner.

Overall mitochondria density was calculated per 15-section series as the sum of mitochondria in presynaptic boutons per 87.25μm^3^ series volume. Likewise, the number of mitochondria within each bouton was counted, and categorized as 0, 1, 2, or 3 or more mitochondria. Finally, mitochondria within each presynaptic bouton were classified and counted within each of three morphological categories: straight, curved, or donut-shaped (toroidal) (Figures 2A–2C). Curved mitochondria had at least one ≤90° bend, donut mitochondria formed ring shapes, and all other mitochondria were classified as straight.

#### Statistical analyses

All statistical analyses were performed separately for CA1 and for dlPFC using the open-source software R.^102^ All analyses used individual animals (cases) as the basic statistical unit, either with a mean measurement per case or by using case as a random effect in multilevel statistical models. All descriptive data are reported as the mean ± SD. We had a limited number of initial exploratory endpoints related to synapse and mitochondrial ultrastructure, and those results guided us to follow-up analyses. For example, the overall effect on synapse area in CA1 guided us to look at the differences between synapse subclasses. In accordance with this process in which initial analyses led us to deeper analyses of significant results, no corrections for multiple comparisons were performed. Exact p values are provided for all analyses. Complete tables of statistical results are included in Tables S1 and S2.

#### Synapse areas, synapse density, vesicle docking, overall mitochondria density

Analyses of synapse areas, measurements of synapse density, vesicle docking ratios, and the overall density of mitochondria in presynaptic boutons were calculated by applying linear mixed models with random effects from the lme4 package^103^ to case-level data, with fixed effects of group, sex, and their interaction, and random effects of case. Additional analysis of synapse area means by quantile was carried out by calculating estimated marginal means of group by quantile and group by sex by quantile. Analyses of atypically large nonperforated synapses defined as ≥2 standard deviations above the mean^65^ were carried out using Fisher’s exact tests comparing numbers and areas of atypically large nonperforated synapses, and all other synapses, between control and anesthesia groups. Homogeneity of variance for synapse density data was evaluated using Bartlett’s test.

#### Counts of mitochondria shapes and numbers per bouton

The counts of: mitochondria classified by shape, the number of mitochondria per bouton across the four numerical categories, and the number of mitochondria per bouton per each count category were analyzed with a hierarchical series of general linear mixed models with random effects using the glmer function of the lme4 package. Poisson distributions were used because the data were counts. Fit of the Poisson distributions to the data was confirmed using the simulateResiduals and testOutliers functions of the DHARMa package.^104^ Group, sex, and group by sex interaction contributions to the observed variance were evaluated using hierarchical Wald chi squared tests.

#### Counts of synapse types

Both a full model of synapse counts across synapse types, classified by postsynaptic target and synapse complexity, and models of synapse counts per each synapse type were analyzed with a hierarchical series of general linear mixed models with random effects using the glmer function of the lme4 package. Poisson distributions were used because the data were counts; fit of the Poisson distributions to the data was confirmed using the simulateResiduals and testOutliers functions of the DHARMa package in R. Group, sex, synapse type, and their interactions’ contributions to the observed variance were evaluated using hierarchical Wald chi squared tests.

#### Areas of synapses by type

A full model of synapse areas sorted by postsynaptic target and synapse complexity was analyzed with a linear mixed model with random effects. Wald chi-squared tests were used to compare a series of hierarchical linear mixed models with random effects to determine group, sex, and group by sex interaction effect contributions to variance across synapse types. Analyses of synapse areas classified by type were carried out using linear mixed models with random effects.

#### Behavioral correlations

Because our sample size is not sufficient for detailed differential analysis of behaviors, we chose to analyze the behavioral and ultrastructural outcomes that were most affected by anesthesia exposures in early life. We chose the human intruder test as a primary behavior for correlation because it showed a consistent impact of anesthesia across life, and was performed closest to the time of brain collection. For each animal, we collated the results of the human intruder test into a composite *Z* score using the behaviors that showed the greatest impact by anesthesia at each age tested. The behaviors most impacted by anesthesia were anxiety behaviors during stare condition at 6 months,^25^ self-directed behaviors during stare at 12 and 24 months,^26^ and freezing during profile condition at 48 months (in prep.). Synapse areas were selected for behavioral correlations because they showed significant impacts of anesthesia. We performed correlation analyses between composite human intruder test z-scores and mean synapse area in CA1, in dlPFC, in quantiles 19 and 20 in CA1 and in dlPFC; mean perforated spinous synapse area in CA1, and mean nonperforated spinous synapse area in dlPFC.

## Data Availability

• Electron microscopy image analysis data have been deposited at https://doi.org/10.5281/zenodo.7272343 and are publicly available as of the date of publication. The DOI is listed in the key resources table. • All original code has been deposited at https://doi.org/10.5281/zenodo.7272343 and is publicly available as of the date of publication. DOIs are listed in the key resources table. • Any additional information required to reanalyze the data reported in this paper is available from the lead contact upon request.
