# Supplementary material for: Neonatal exposures to sevoflurane in rhesus monkeys alter synaptic ultrastructure in later life
Source: iScience. 2022 Nov 30;25(12):105685. doi: 10.1016/j.isci.2022.105685 (PMC9772858; doi:10.1016/j.isci.2022.105685)
Supplement: Document S1. Tables S1 and S2 [file mmc1.pdf]

iScience, Volume *25*

Supplemental information

Neonatal exposures to sevoflurane in rhesus monkeys alter synaptic ultrastructure in later life

Tristan Fehr, William G.M. Janssen, Janis Park, and Mark G. Baxter

Supplemental table 1: Statistical summaries of synapse analyses, related to Figures 3-4.

	anesthesia group	sex	anesthesia x sex
CA1 area (**Fig. 3A**)	F(1, 13.98) = 10.68, p = 0.01	F(1, 13.98) = 2, p = 0.18	F(1, 13.98) = 0.33, p = 0.58
CA1 density (**Fig. 3E**)	F(1, 14) = 0.06, p = 0.81	F(1, 14) = 5.95, p = 0.03	F(1, 14) = 0.23, p = 0.64
CA1 vesicles (**Fig. 3F**)	F(1, 14) = 0.88, p = 0.36	F(1, 14) = 2.03, p = 0.18	F(1, 14) = 0.02, p = 0.9

	anesthesia group	sex	anesthesia x sex
dlPFC area (**Fig. 3C**)	F(1, 14.06) = 1.96, p = 0.18	F(1, 14.06) = 0.37, p = 0.55	F(1, 14.06) = 0, p = 0.95
dlPFC density (**Fig. 3G**)	F(1, 14) = 0.53, p = 0.48	F(1, 14) = 1.5, p = 0.24	F(1, 14) = 0, p = 1
dlPFC vesicles (**Fig. 3H**)	F(1, 13.98) = 0.45, p = 0.51	F(1, 13.98) = 0.74, p = 0.4	F(1, 13.98) = 0.44, p = 0.52

CA1 area by quantile (**Fig. 3B**)	
group	F(1, 14) = 10.2, p = 0.01
sex	F(1, 14) = 1.44, p = 0.25
quantile	F(19, 266) = 537.15, p < 0.0005
group x sex	F(1, 14) = 2.03, p = 0.18
group x quantile	F(19, 266) = 1.27, p = 0.2
sex x quantile	F(19, 266) = 0.44, p = 0.98
group x sex x quantile	F(19, 266) = 2.77, p < 0.0005

dlPFC area by quantile (**Fig. 3D**)	
group	F(1, 14) = 3.56, p = 0.08
sex	F(1, 14) = 0.35, p = 0.56
quantile	F(19, 266) = 638.82, p < 0.0005
group x sex	F(1, 14) = 0.05, p = 0.83
group x quantile	F(19, 266) = 2.81, p < 0.0005
sex x quantile	F(19, 266) = 0.09, p = 1
group x sex x quantile	F(19, 266) = 0.49, p = 0.97

CA1 overall synapse type counts (**Fig. 4A,C**)	
sex	$\chi^2(1) = 0.551$, $p = 0.458$
group	$\chi^2(1) = 0.071$, $p = 0.79$
group x sex	$\chi^2(1) = 0.108$, $p = 0.742$
synapse type†	$\chi^2(3) = 9658.611$, $p < 0.0005$
sex x synapse type	$\chi^2(3) = 10.402$, $p = 0.015$
group x synapse type	$\chi^2(3) = 33.887$, $p < 0.0005$
group x sex x synapse type	$\chi^2(3) = 7.595$, $p = 0.055$

† type = factor with 4 levels: perforated spinous, perforated dendritic, nonperforated spinous, nonperforated dendritic

**Fig. 4A,C**	anesthesia group	sex	anesthesia x sex
CA1 perforated spinous count	$\chi^2(1) = 0.415$, $p = 0.519$	$\chi^2(1) = 0.477$, $p = 0.49$	$\chi^2(1) = 1.147$, $p = 0.284$
CA1 perforated dendritic count	$\chi^2(1) = 6.113$, $p = 0.013$	$\chi^2(1) = 0.683$, $p = 0.408$	$\chi^2(1) = 0.018$, $p = 0.894$
CA1 nonperforated spinous count	$\chi^2(1) = 0.001$, $p = 0.972$	$\chi^2(1) = 0.654$, $p = 0.419$	$\chi^2(1) = 0.556$, $p = 0.456$
CA1 nonperforated dendritic count	$\chi^2(1) = 6.017$, $p = 0.014$	$\chi^2(1) = 1.495$, $p = 0.221$	$\chi^2(1) = 0.081$, $p = 0.775$

dlPFC overall synapse type counts (**Fig. 4B,D**)	
sex	$\chi^2(1) = 2.958$, $p = 0.085$
group	$\chi^2(1) = 1.534$, $p = 0.215$
group x sex	$\chi^2(1) = 4.244$, $p = 0.039$
synapse type	$\chi^2(3) = 5004.244$, $p < 0.0005$
sex x synapse type	$\chi^2(3) = 4.054$, $p = 0.256$
group x synapse type	$\chi^2(3) = 18.251$, $p < 0.0005$
group x sex x synapse type	$\chi^2(3) = 2.056$, $p = 0.561$

**Fig. 4B,D**	anesthesia group	sex	anesthesia x sex
dlPFC perforated spinous count	$\chi^2(1) = 1.242$, $p = 0.265$	$\chi^2(1) = 0.304$, $p = 0.581$	$\chi^2(1) = 0.251$, $p = 0.616$
dlPFC perforated dendritic count	$\chi^2(1) = 0.217$, $p = 0.641$	$\chi^2(1) = 2.518$, $p = 0.113$	$\chi^2(1) = 0.3$, $p = 0.584$
dlPFC nonperforated spinous count	$\chi^2(1) = 2.541$, $p = 0.111$	$\chi^2(1) = 1.133$, $p = 0.287$	$\chi^2(1) = 1.781$, $p = 0.182$
dlPFC nonperforated dendritic count	$\chi^2(1) = 1.131$, $p = 0.288$	$\chi^2(1) = 0.002$, $p = 0.964$	$\chi^2(1) = 0.897$, $p = 0.344$

CA1 area by type (**Fig. 4E,F**)	
sex x type	$\chi^2(3) = 7.455, p = 0.059$
group x type	$\chi^2(3) = 8.712, p = 0.033$
group x sex x type	$\chi^2(3) = 18.969, p < 0.0005$

**Fig. 4E,F**	anesthesia group	sex	anesthesia x sex
CA1 perforated spinous area	F(1, 11.8) = 5.19, p = 0.04	F(1, 11.8) = 0.9, p = 0.36	F(1, 11.8) = 0.48, p = 0.5
CA1 perforated dendritic area	F(1, 14.8) = 0.03, p = 0.88	F(1, 14.8) = 0.03, p = 0.86	F(1, 14.8) = 2.86, p = 0.11
CA1 nonperforated spinous area	F(1, 14.07) = 0.24, p = 0.63	F(1, 14.07) = 0.05, p = 0.82	F(1, 14.07) = 0.94, p = 0.35
CA1 nonperforated dendritic area	F(1, 13.85) = 0.21, p = 0.65	F(1, 13.85) = 0.13, p = 0.73	F(1, 13.85) = 0.58, p = 0.46

dlPFC area by type (**Fig. 4G,H**)	
sex x type	$\chi^2(3) = 1.36, p = 0.715$
group x type	$\chi^2(3) = 4.403, p = 0.221$
group x sex x type	$\chi^2(3) = 8.768, p = 0.033$

**Fig. 4G,H**	anesthesia group	sex	anesthesia x sex
dlPFC perforated spinous area	F(1, 15.73) = 0.02, p = 0.9	F(1, 15.73) = 0.39, p = 0.54	F(1, 15.73) = 3.13, p = 0.1
dlPFC perforated dendritic area	F(1, 91) = 0.32, p = 0.57	F(1, 91) = 0.05, p = 0.83	F(1, 91) = 0.45, p = 0.5
dlPFC nonperforated spinous area	F(1, 14.89) = 6.66, p = 0.02	F(1, 14.89) = 3.69, p = 0.07	F(1, 14.89) = 0.81, p = 0.38
dlPFC nonperforated dendritic area	F(1, 14.49) = 0.17, p = 0.69	F(1, 14.49) = 0.74, p = 0.4	F(1, 14.49) = 0.16, p = 0.69

Behavioral correlations

CA1 mean synapse area with behavioral z-score	r = -0.539, p = .021
DLPFC mean synapse area with behavioral z-score	r = -0.347, p = .158
CA1 19th quantile area with behavioral z-score	r = -0.354, p = .150
CA1 20th quantile area with behavioral z-score	r = -0.099, p = .696
DLPFC 19th quantile with behavioral z-score	r = -0.185, p = .464
DLPFC 20th quantile with behavioral z-score	r = -0.298, p = .230

Supplemental table 2: Statistical summaries of mitochondrial analyses, related to Figures 5-6.

	anesthesia group	sex	anesthesia x sex
CA1 mitochondria density (**Fig. 5A**)	$F(1, 14) = 0.72, p = 0.41$	$F(1, 14) = 0.61, p = 0.45$	$F(1, 14) = 2.87, p = 0.11$
dlPFC mitochondria density (**Fig. 5C**)	$F(1, 14) = 1.1, p = 0.31$	$F(1, 14) = 0.39, p = 0.54$	$F(1, 14) = 1.18, p = 0.3$

CA1 mitochondria shape (**Fig. 5B**)	anesthesia group	sex	anesthesia x sex
CA1 straight	$\chi^2(1) = 0.289, p = 0.591$	$\chi^2(1) = 0.746, p = 0.388$	$\chi^2 1) = 1.116, p = 0.291$
CA1 curved	$\chi^2(1) = 4.121, p = 0.042$	$\chi^2(1) = 1.524, p = 0.217$	$\chi^2(1) = 0.001, p = 0.973$
CA1 toroid	$\chi^2(1) = 0.499, p = 0.48$	$\chi^2(1) = 0.494, p = 0.482$	$\chi^2(1) = 2.06, p = 0.151$

dlPFC mitochondria shape (**Fig. 5D**)	anesthesia group	sex	anesthesia x sex
dlPFC straight	$\chi^2(1) = 0.333, p = 0.564$	$\chi^2(1) = 0.151, p = 0.698$	$\chi^2(1) = 2.247, p = 0.134$
dlPFC curved	$\chi^2(1) = 1.322, p = 0.25$	$\chi^2(1) = 0.172, p = 0.678$	$\chi^2(1) = 0.719, p = 0.396$
dlPFC toroid	$\chi^2(1) = 1.589, p = 0.208$	$\chi^2(1) = 0.306, p = 0.58$	$\chi^2(1) = 1.421, p = 0.233$

overall CA1 number of boutons by frequency of mitochondria (0 / 1 / 2 / 3 or more) (**Fig. 6A**)	
sex	$\chi^2(1) = 0.636, p = 0.425$
group	$\chi^2(1) = 0.589, p = 0.443$
group x sex	$\chi^2(1) = 2.871, p = 0.09$
bouton freq	$\chi^2(3) = 4337.328, p < 0.0005$
sex x bouton freq	$\chi^2(3) = 2.766, p = 0.429$
group x bouton freq	$\chi^2(3) = 4.386, p = 0.223$
group x sex x bouton freq	$\chi^2(3) = 5.18, p = 0.159$

**Fig. 6A**	anesthesia group	sex	anesthesia x sex
CA1 0 mito boutons	$\chi^2(1) = 0.159, p = 0.69$	$\chi^2(1) = 0.529, p = 0.467$	$\chi^2(1) = 3.192, p = 0.074$
CA1 1 mito boutons	$\chi^2(1) = 1.6, p = 0.206$	$\chi^2(1) = 0.586, p = 0.444$	$\chi^2(1) = 2.518, p = 0.113$
CA1 2 mito boutons	$\chi^2(1) = 0.054, p = 0.816$	$\chi^2(1) = 0.203, p = 0.653$	$\chi^2(1) = 0.377, p = 0.539$
CA1 3+ mito boutons	$\chi^2(1) = 2.099, p = 0.147$	$\chi^2(1) = 3.721, p = 0.054$	$\chi^2(1) = 1.336, p = 0.248$

overall dlPFC number of boutons by frequency of mitochondria (0 / 1 / 2 / 3 or more) (**Fig. 6B**)	
sex	$\chi^2(1) = 0.019, p = 0.89$
group	$\chi^2(1) = 0.214, p = 0.644$
group x sex	$\chi^2(1) = 6.135, p = 0.013$
bouton freq	$\chi^2(3) = 3680.777, p < 0.0005$
sex x bouton freq	$\chi^2(3) = 2.36, p = 0.501$
group x bouton freq	$\chi^2(3) = 4.131, p = 0.248$
group x sex x bouton freq	$\chi^2(3) = 1.834, p = 0.608$

**Fig. 6B**	anesthesia group	sex	anesthesia x sex
dlPFC 0 mito boutons	$\chi^2(1) = 0.159, p = 0.69$	$\chi^2(1) = 0.022, p = 0.882$	$\chi^2(1) = 4.151, p = 0.042$
dlPFC 1 mito boutons	$\chi^2(1) = 1.448, p = 0.229$	$\chi^2(1) = 0.073, p = 0.787$	$\chi^2(1) = 2.911, p = 0.088$
dlPFC 2 mito boutons	$\chi^2(1) = 0.386, p = 0.534$	$\chi^2(1) = 1.472, p = 0.225$	$\chi^2(1) = 0.803, p = 0.37$
dlPFC 3+ mito boutons	$\chi^2(1) = 1.441, p = 0.23$	$\chi^2(1) = 0.07, p = 0.792$	$\chi^2(1) = 2.338, p = 0.126$
